# Supplementary material for: A biobank-scale test of marginal epistasis reveals genome-wide signals of polygenic interaction effects
Source: Nat Genet. 2025 Dec 9;57(12):3175–84. doi: 10.1038/s41588-025-02411-y (PMC12695669; doi:10.1038/s41588-025-02411-y)
Supplement: Supplementary file 1 — Supplementary Notes 1–8, Figs. 1–14, Tables 1–10 and References. [file 41588_2025_2411_MOESM1_ESM.pdf]

nature genetics

Supplementary information https://doi.org/10.1038/s41588-025-02411-y

A biobank-scale test of marginal epistasis reveals genome-wide signals of polygenic interaction effects

In the format provided by the authors and unedited

Supplementary Information

Supplementary Notes

1 Assessing the impact of model misspecification

***UKBB-small*:** For these experiments, we used a subset of the UKBB array genotypes obtained by restricting to SNPs on chromosomes 12 and 20 across $291,273$ unrelated white British individuals. We excluded SNPs with MAF less than 1% resulting in a final set of $32,708$ SNPs. We term this dataset "*UKBB-small*". For simulations that involve feature missingness, we constructed imputed genotypes by restricting to SNPs on chromosomes 12 and 20 resulting in 340,776 SNPs. We term this dataset "*UKBB-small-imputed*". Experiments that attempt to assess the impact of model mis-specification typically use the *UKBB-small* dataset. In cases where the simulation involves assessing the impact of unobserved SNPs, the *UKBB-small-imputed* dataset is used.

Polynomial covariate relationship

This experiment aims to simulate a scenario in which the impact of covariates $\boldsymbol{C}$ on the phenotype $\boldsymbol{y}$ is described by a nonlinear (quadratic) function. However, this polynomial relationship is not known. As a result, when we apply FAME, we only include the original untransformed covariates.

The generative model for this setting is as follows:

$$
\begin{aligned}
\boldsymbol{y} &= \mathbf{X}\boldsymbol{\beta} + \Phi(\mathbf{C})\boldsymbol{\gamma} + \boldsymbol{\epsilon} \\
\boldsymbol{\epsilon} &\sim \mathcal{N}(\mathbf{0}, \sigma_e^2 \boldsymbol{I}_N) \\
\boldsymbol{\beta} &\sim \mathcal{N}(\mathbf{0}, \frac{\sigma_g^2}{M} \boldsymbol{I}_M) \\
\boldsymbol{\gamma} &\sim \mathcal{N}(\mathbf{0}, \frac{\sigma_{cxc}^2}{D} \boldsymbol{I}_D)
\end{aligned}
$$

Here $\mathbf{X}$ is the genotype matrix, Φ represents the second-order polynomial transformation (of dimension D), and $\mathbf{C}$ represents the set of covariates (we use age and sex). σ_{cxc}^2 represents the variance explained by the transformed covariates, σ_g^2 represents the variance explained by additive effects, and σ_ϵ^2 represents the noise variance. In this experiment, we simulated phenotypes under two parameter settings (σ_g^2, σ_{cxc}^2, σ_ϵ^2) = (0.1,0.25,0.9) and (0.05,0.25,0.95).

G-E with unobserved E

Unknown interactions can affect the model calibration. Such variables can be unobserved environmental variables, missing-tagged causal SNPs, or aggregated PGS.

The generative model for this setting is as follows:

$$
\begin{aligned}
&\boldsymbol{y} = \mathbf{X}\boldsymbol{\beta} + \sum_{j \in S} \boldsymbol{\omega}_j \mathbf{X}_{:,j} \odot \mathbf{h} + \boldsymbol{\epsilon} \\
&\boldsymbol{\omega} \sim \mathcal{N}(\mathbf{0}, \frac{\sigma_{hid}^2}{|S|} \boldsymbol{I}) \\
&\boldsymbol{\epsilon} \sim \mathcal{N}(\mathbf{0}, \sigma_\epsilon^2 \boldsymbol{I})
\end{aligned}
$$

Here $\mathbf{h}$ is an unobserved environmental variable (E) drawn from a standard Gaussian distribution and σ_{hid}^2

denotes the variance explained by the hidden G-E. S represents the subset of SNPs selected to interact with the hidden E. In this experiment, we randomly select 10% of the observed SNPs to interact with the hidden E. We further simulated under two parameter settings (σ_g^2, σ_{hid}^2, σ_ϵ^2) = (0.1,0.5,0.9) and (0.05,0.5,0.95).

Heavy-tailed noise

Here we considers the setting where the phenotype has an additive architecture while the environmental noise is drawn from a long-tailed distribution. To achieve this, we simulate the noise using the Student's t-distribution from the following generative model:

$$\begin{aligned}\boldsymbol{y} &= \mathbf{X}\boldsymbol{\beta} + \boldsymbol{\epsilon}\\ \boldsymbol{\beta} &\sim \mathcal{N}(\mathbf{0}, \frac{\sigma_g^2}{M}\boldsymbol{I})\\ \epsilon_n &\overset{iid}{\sim} \sqrt{s_\epsilon}t_\nu\end{aligned}$$

Here t_ν is the t-distribution with degree of freedom ν (with variance $\frac{\nu}{\nu-2}$ for $\nu > 2$) and s_ϵ is the scaling parameter. To simulate from a heavy-tailed distribution, we set $\nu = 3$ and $3s_\epsilon = \sigma_\epsilon^2$ to ensure a specific environmental variance (σ_ϵ^2). In this experiment, we simulated two settings (σ_g^2, σ_ϵ^2) = (0.1, 0.9) and (0.05, 0.95).

Heteroskedastic noise

We assesses the impact of heteroskedastic noise using the following generative model:

$$\begin{aligned}\boldsymbol{y} &= \mathbf{X}\boldsymbol{\beta} + \mathbf{z} \odot \boldsymbol{\delta} + \boldsymbol{\epsilon}\\ \boldsymbol{\beta} &\sim \mathcal{N}(\mathbf{0}, \frac{\sigma_g^2}{M}\boldsymbol{I})\\ \boldsymbol{\delta} &\sim \mathcal{N}(\mathbf{0}, \sigma_{het}^2\boldsymbol{I})\\ \boldsymbol{\epsilon} &\sim \mathcal{N}(\mathbf{0}, \sigma_\epsilon^2\boldsymbol{I})\end{aligned}$$

Hhere $\boldsymbol{z}$ is a binary indicator variable drawn from $Bern(0.5)$, denoting which samples to assign to the group with larger variance. In this experiment, we simulated phenotypes under two parameter settings (σ_g^2, σ_{het}^2, σ_ϵ^2) = (0.1,0.5,0.9) and (0.05,0.5,0.95).

Large additive effects

We examined whether FAME is robust in the presence of genetic variants with large additive effects by considering the following generative model:

$$
\begin{aligned}
&\boldsymbol{y} = \mathbf{X}\boldsymbol{\beta} + \sum_{j \in S} \boldsymbol{\omega}_j \mathbf{X}_{:,j} + \boldsymbol{\epsilon} \\
&\beta_m \overset{iid}{\sim} 0.1\mathcal{N}(\mathbf{0}, \frac{\sigma_g^2}{0.1 \times M}\boldsymbol{I}) + 0.9\delta_0 \\
&\boldsymbol{\omega} \sim \mathcal{N}(\mathbf{0}, \frac{\sigma_{large}^2}{|S|}\boldsymbol{I}) \\
&\boldsymbol{\epsilon} \sim \mathcal{N}(\mathbf{0}, \sigma_\epsilon^2 \boldsymbol{I})
\end{aligned}
$$

We first simulated the additive effect by randomly selecting 10% of SNPs from *UKBB-small*, where the effect sizes were drawn from an i.i.d. normal distribution. Additionally, we identified a distinct set, S, comprising 0.1% of SNPs, where the SNPs were drawn from a distribution with a substantially higher variance thereby endowing each SNP in set S with the potential for a large marginal effect. Specifically, we simulated two settings (σ_g^2, σ_{large}^2, σ_ϵ^2) = (0.1,0.1,0.9) and (0.05,0.1,0.95).

Imperfectly-tagged large additive effects

This experiment is similar to the Large additive effects setting (Section 1) except that we relaxed the assumption that all the extremely large effect variants were observed, acknowledging the presence of imperfectly tagged SNPs. Specifically, we simulated phenotypes using the imputed SNPs (*UKBB-small-imputed*) while during the estimation stage, we assumed only the SNPs in *UKBB-small* array data were observed.

Imperfectly-tagged LDAK model

This experiment aims to study the calibration of FAME when the true genetic architecture has a different relationship between effect size, MAF, and LD (LDAK model [1]) than the one assumed in FAME. In addition, we assume that not all causal SNPs are observed.

$$
\begin{aligned}
&\boldsymbol{y} = \mathbf{X}\boldsymbol{\beta} + \boldsymbol{\epsilon} \\
&\boldsymbol{\beta} \sim \mathcal{N}(\mathbf{0}, diag(\sigma_{g1}^2, .., \sigma_{gM}^2)) \\
&\boldsymbol{\epsilon} \sim \mathcal{N}(\mathbf{0}, \sigma_\epsilon^2 \boldsymbol{I})
\end{aligned}
$$

We assumed the LDAK genetic architecture. Specifically, $\sigma_{gm}^2 = Sc_m w^b [f_m(1-f_m)]^a$, where S is the normalization constant such that $\sum_{m=1}^{M} \sigma_{gm}^2 = \sigma_g^2$; c_m is an indicator variable indicating whether SNP m is causal; f_m is the MAF of SNP m; w_m is the LD score of SNP m, and $a = 0.75$, $b = 1$ for the LDAK model. Further, we simulated the phenotype using imputed genotypes (*UKBB-small-imputed*). However, for estimation, we used the array version of *UKBB-small*, a subset of the imputed version, to mimic scenarios with missing-causal SNPs. We set $(\sigma_g^2, \sigma_\epsilon^2) = (0.05, 0.95)$ and $(0.1, 0.9)$, the causal SNP ratio was set to 0.1.

2 Estimating the standard error of $\hat{\sigma}^2_{gxg,t}/\hat{\sigma}^2_{gwas,t}$

We applied error propagation to compute the standard error of $\sigma^2_{gxg,t}/\sigma^2_{gwas,t}$. First, let's define $f(\sigma^2_{gwas,t}, \sigma^2_{gxg,t}) \equiv \frac{\sigma^2_{gxg,t}}{\sigma^2_{gwas,t}}$, then the standard error of $f(\sigma^2_{gwas,t}, \sigma^2_{gxg,t})$ is

$$\begin{aligned} Var(f(\sigma^2_{gwas,t}, \sigma^2_{gxg,t})) &= (\frac{\partial f}{\partial \sigma^2_{gwas,t}})^2 Var(\sigma^2_{gwas,t}) + (\frac{\partial f}{\partial \sigma^2_{gxg,t}})^2 Var(\sigma^2_{gxg,t}) \\ &= Var(\sigma^2_{gwas,t})\Big(-\frac{\sigma^2_{gxg,t}}{(\sigma^2_{gwas,t})^2}\Big)^2 + Var(\sigma^2_{gxg,t})\Big(\frac{1}{\sigma^2_{gwas,t}}\Big)^2 \end{aligned}$$

Therefore

$$SE(f(\sigma^2_{gwas,t}, \sigma^2_{gxg,t})) = \frac{\sqrt{Var(\sigma^2_{gwas,t})(\sigma^2_{gxg,t})^2 + Var(\sigma^2_{gxg,t})(\sigma^2_{gwas,t})^2}}{(\sigma^2_{gwas,t})^2}$$

3 Robustness of significant marginal epistasis signals

Population stratification: Population stratification in GWAS is commonly accounted for by including principal components (PCs) computed from genotype data as covariates in the analysis [2, 3]. To explore the effect of population stratification, we reran our analyses on trait-SNP pairs previously discovered as significant with the number of PCs included as covariates increased to 40 (from 20). We observe a high concordance of the estimates when using 40 vs 20 PCs (Supplementary Figure 10).

Imperfectly tagged causal SNPs: A second concern with our analyses arises from the fact that the UK Biobank array might miss true causal variants which could lead to the inference of spurious epistatic effects [4, 5, 6]. Our simulations in Section 'Calibration of FAME' show that FAME remains calibrated in this setting. To further explore the robustness of our results, we analyzed our significant marginal epistasis signals on $4,824,392$ imputed SNPs (MAF $> 1\%$). We observed concordance of the estimates on the imputed dataset with those obtained on the array dataset (Supplementary Figure 10) with the two SNPs for SHBG showing significantly lower marginal epistasis estimates.

Permutation tests to assess estimates of marginal epistasis: To assess whether the identified marginal epistasis significant trait-SNP pairs are influenced by inflation due to extraneous factors, we performed a permutation of the epistatic matrix relative to the target variant. This was done while preserving the underlying additive structure. Specifically, we constructed a permuted marginal epistasis matrix, denoted as $\boldsymbol{E}_{t(\text{perm})} = \boldsymbol{X}_{-t} \odot \boldsymbol{X}_{:t(\text{perm})}$, keeping the additive matrix $\boldsymbol{X}$ intact. This approach ensured that all additive effects, including those of the target variant, remained unaltered. Our observations revealed that, following this permutation, none of the previously detected trait-SNP pairs met the significance thresholds (detailed in the Supplementary Table 4).

Impact of phenotype scale: The scale on which the phenotype is measured can affect approaches to test and interpret epistasis [7]. To explore the impact of scale, we performed null simulations where the phenotype has an additive genetic architecture but no genetic interactions. Next, we considered transformations of the phenotype: $sign(y) * |y|^s$, where s is a scaling factor. We randomly selected 200 LD-pruned GWAS hits and applied FAME to estimate marginal epistasis at these SNPs. The figure shows the inflation of the p-values estimated by FAME with increasing magnitude of the scaling factor ($s = 1$ is the original unscaled phenotype; Supplementary Figure 11). In all our analyses, traits are inverse rank normalized so that the results of FAME are invariant to monotone transformations (such as the scaling transformation considered here) and the p-values remain calibrated, as we confirm in simulations (Supplementary Figure 11).

4 Estimating heritability from variance component estimates

Here we compute the heritability explained by the marginal epistasis variance component estimated in Equation 5 following the derivation in [1].

Under the assumption that all matrices are standardized, the heritability associated with marginal epistasis effects at SNP t can be computed as:

$$\hat{h}^2_{gxg,t} = \frac{\hat{\sigma}^2_{gxg,t}}{\hat{\sigma}^2_g + \hat{\sigma}^2_{gxg,t} + \hat{\sigma}^2_e}$$

However, if the matrix $\mathbf{E} = \mathbf{X}_{-t} \odot \mathbf{X}_t$ is not standardized, then we need to derive a more general formula.

Let V_T denote the total phenotypic variance.

$$V_T = \frac{\sum_i y_i^2}{N} - \Big(\frac{\sum_i y_i}{N}\Big)^2 \tag{8}$$

We can re-write our model as follows:

$$y_i = \sum_{j=1}^{M} x_{ij}\beta_j + \sum_{k=1}^{M-1} e_{ik}\alpha_k + \epsilon_i$$

Here e_{ik} is the element in the ith row and k^{th} column of $\boldsymbol{E}$. Based on the distributional assumptions of our model, the following holds:

$$\begin{aligned}
\mathbb{E}[\beta_j\beta_{j'}] = 0 \text{ for } j \neq j', \quad \mathbb{E}[\beta_j^2] = \frac{\sigma_g^2}{M} \\
\mathbb{E}[\alpha_k\alpha_{k'}] = 0 \text{ for } k \neq k', \quad \mathbb{E}[\alpha_k^2] = \frac{\sigma^2_{gxg,t}}{M'} \\
\mathbb{E}[\epsilon_i\epsilon_{i'}] = 0 \text{ for } i \neq i', \quad \mathbb{E}[\epsilon_i^2] = \sigma_\epsilon^2 \\
\mathbb{E}[\beta_j\alpha_k] = 0; \quad \mathbb{E}[\beta_j\epsilon_i] = 0; \quad \mathbb{E}[\alpha_k\epsilon_i] = 0;
\end{aligned}$$

where $M' = M - 1$.

To compute the expectation of the terms in the first summation of Equation 8:

$$\begin{aligned}
\mathbb{E}[y_i^2] &= \mathbb{E}[(\sum_{j=1}^{M} x_{ij}\beta_j + \sum_{k=1}^{M'} e_{ik}\alpha_k + \epsilon_i)^2] \\
&= \sum_{j=1}^{M} x_{ij}^2 \mathbb{E}[\beta_{ij}^2] + \sum_{k=1}^{M'} e_{ik}^2 \mathbb{E}[\alpha_k^2] + \mathbb{E}[\epsilon_i^2] \\
&= (\sum_{j=1}^{M} x_{ij}^2)\frac{\sigma_g^2}{M} + (\sum_{k=1}^{M'} e_{ik}^2)\frac{\sigma^2_{gxg,t}}{M'} + \sigma_\epsilon^2
\end{aligned}$$

This gives us:

$$\frac{1}{N}\sum_i \mathbb{E}[y_i^2] = \frac{1}{N}(\sum_{i=1}^{N}\sum_{j=1}^{M} x_{ij}^2)\frac{\sigma_g^2}{M} + \frac{1}{N}(\sum_{i=1}^{N}\sum_{k=1}^{M'} e_{ik}^2)\frac{\sigma_{gxg,t}^2}{M'} + \sigma_\epsilon^2$$
$$= \frac{tr(\boldsymbol{X}\boldsymbol{X}^T)}{N}\frac{\sigma_g^2}{M} + \frac{tr(\boldsymbol{E}\boldsymbol{E}^T)}{N}\frac{\sigma_{gxg,t}^2}{M'} + \sigma_\epsilon^2 \tag{9}$$

The expected value for the second summation in Equation 8 is:

$$\mathbb{E}[(y_1 + ... + y_N)^2] \tag{10}$$
$$= \mathbb{E}[\sum_{ii'} x_{i1}x_{i'1}\beta_1^2 + ... + \sum_{ii'} x_{iM}x_{i'M}\beta_M^2 + \sum_{ii'} e_{i1}e_{i'1}\alpha_1^2 + ... + \sum_{ii'} e_{iM'}e_{i'M'}\alpha_{M'}^2 + \sum_i \epsilon_i^2]$$
$$= (\sum_j\sum_{ii'} x_{ij}x_{i'j})\frac{\sigma_g^2}{M} + (\sum_k\sum_{ii'} e_{ik}e_{i'k})\frac{\sigma_{gxg,t}^2}{M'} + N\sigma_\epsilon^2$$
$$= sum(\boldsymbol{X}\boldsymbol{X}^T)\frac{\sigma_g^2}{M} + sum(\boldsymbol{E}\boldsymbol{E}^T)\frac{\sigma_{gxg,t}^2}{M'} + N\sigma_\epsilon^2 \tag{11}$$

Therefore

$$\mathbb{E}[V_T] = \frac{tr(\boldsymbol{X}\boldsymbol{X}^T)}{N}\frac{\sigma_g^2}{M} + \frac{tr(\boldsymbol{E}\boldsymbol{E}^T)}{N}\frac{\sigma_{gxg,t}^2}{M'} + \sigma_\epsilon^2 - \frac{sum(\boldsymbol{X}\boldsymbol{X}^T)}{N^2}\frac{\sigma_g^2}{M} - \frac{sum(\boldsymbol{E}\boldsymbol{E}^T)}{N^2}\frac{\sigma_{gxg,t}^2}{M'} - \frac{1}{N}\sigma_\epsilon^2$$
$$= \sigma_g^2 + \left(\frac{tr(\boldsymbol{E}\boldsymbol{E}^T)}{N} - \frac{sum(\boldsymbol{E}\boldsymbol{E}^T)}{N^2}\right)\frac{\sigma_{gxg,t}^2}{M'} + \frac{N-1}{N}\sigma_\epsilon^2$$

where the last equation holds under the assumption that $\boldsymbol{X}$ is standardized.

We define the residual with respect to the marginal epistasis variance component as $r_i = \sum_{j=1}^{M} x_{ij}\beta_j + \epsilon_i$ so that the residual variance is:

$$\mathbb{E}[V_R] = \frac{tr(\boldsymbol{X}\boldsymbol{X}^T)}{N}\frac{\sigma_g^2}{M} + \sigma_\epsilon^2 - \frac{sum(\boldsymbol{X}\boldsymbol{X}^T)}{N^2}\frac{\sigma_g^2}{M} - \frac{1}{N}\sigma_\epsilon^2$$
$$= \sigma_g^2 + \frac{N-1}{N}\sigma_\epsilon^2$$

Finally, the expected marginal epistasis heritability can be computed as

$$\mathbb{E}[h_{gxg,t}^2] \approx 1 - \frac{\mathbb{E}[V_R]}{\mathbb{E}[V_T]}$$
$$= 1 - \frac{\sigma_g^2 + \frac{N-1}{N}\sigma_\epsilon^2}{\sigma_g^2 + (\frac{tr(\boldsymbol{E}\boldsymbol{E}^T)}{N} - \frac{sum(\boldsymbol{E}\boldsymbol{E}^T)}{N^2})\frac{\sigma_{gxg,t}^2}{M'} + \frac{N-1}{N}\sigma_\epsilon^2}$$
$$= \frac{(\frac{tr(\boldsymbol{E}\boldsymbol{E}^T)}{N} - \frac{sum(\boldsymbol{E}\boldsymbol{E}^T)}{N^2})\frac{\sigma_{gxg,t}^2}{M'}}{\sigma_g^2 + (\frac{tr(\boldsymbol{E}\boldsymbol{E}^T)}{N} - \frac{sum(\boldsymbol{E}\boldsymbol{E}^T)}{N^2})\frac{\sigma_{gxg,t}^2}{M'} + \frac{N-1}{N}\sigma_\epsilon^2}$$
$$= \frac{\mathcal{C}\sigma_{gxg,t}^2}{\sigma_g^2 + \mathcal{C}\sigma_{gxg,t}^2 + \frac{N-1}{N}\sigma_\epsilon^2}$$

where $\mathcal{C} \equiv (\frac{tr(\boldsymbol{E}\boldsymbol{E}^T)}{N} - \frac{sum(\boldsymbol{E}\boldsymbol{E}^T)}{N^2})\frac{1}{M'}$.

We plug-in the estimates $\tilde{\boldsymbol{\sigma}}^2$ to obtain

$$\tilde{h}^2_{gxg,t} = \frac{\tilde{\mathcal{C}}\tilde{\sigma}^2_{gxg,t}}{\tilde{\sigma}^2_g + \tilde{\mathcal{C}}\tilde{\sigma}^2_{gxg,t} + \frac{N-1}{N}\tilde{\sigma}^2_\epsilon}$$

where $\tilde{\mathcal{C}}$ is computed efficiently using the randomized trace estimates.
During the actual computation, $tr(\boldsymbol{E}\boldsymbol{E}^T)$ can be directly accessed via the model intermediate output while we compute $sum(\boldsymbol{E}\boldsymbol{E}^T)$ as:

$$\begin{aligned}
sum(\boldsymbol{E}\boldsymbol{E}^T) &= \sum_{ii'}(\boldsymbol{E}\boldsymbol{E}^T)_{ii'} \\
&= \sum_{ii'}\sum_j e_{ij}e_{i'j} \\
&= \sum_j\sum_{ii'} e_{ij}e_{i'j} \\
&= \sum_j(\sum_i e_{ij})(\sum_{i'} e_{i'j}) \\
&= \sum_j(\sum_i e_{ij})^2 \\
&= N^2\sum_j corr(\boldsymbol{X}_{:t}, \boldsymbol{X}_{:j})^2
\end{aligned}$$

where the last equation holds because

$$\begin{aligned}
e_{ij} &= x_{it}x_{ij} \\
\sum_i e_{ij} &= \sum_i x_{it}x_{ij} \\
&= NCorr(\boldsymbol{X}_{:t}, \boldsymbol{X}_{:j})
\end{aligned} \tag{12}$$

and Equation 12 holds under the assumption that $\mathbf{X}$ is column-wise standardized. In practice, we estimate the squared correlation with Plink [8].

To further estimate the standard error of $h^2_{gxg,t}$, we use the fact that the target traits have been standardized; therefore, $V_T \approx 1$, and thus

$$\begin{aligned}
Var(h^2_{gxg,t}) &= Var(1 - \frac{\mathbb{E}[V_R]}{\mathbb{E}[V_T]}) \\
&\approx Var(1 - \mathbb{E}[V_R]) \\
&= Var(\mathbb{E}[V_R]) \\
&= Var(\mathcal{C}\sigma^2_{gxg,t}) \\
&= \mathcal{C}^2 Var(\sigma^2_{gxg,t})
\end{aligned}$$

Therefore the corresponding estimator for standard error $\widetilde{SE}(h^2_{gxg,t}) = \mathcal{C} * \widetilde{SE}(\sigma^2_{gxg,t})$.

5 Interpretation of individual marginal epistasis loci

Lipoprotein A: We observe the largest magnitude of h^2_{gxg} and the largest ratio of h^2_{gxg} to h^2_{gwas} at SNP rs628031 for serum lipoprotein A levels (lipoA). This variant lies in a locus (near the *LPA* gene) that has been shown to explain as much as 90% of trait variance [9]. rs628031 is a non-synonymous polymorphism in the protein product of the *OCT1* gene (also known as *SLC22A1*). *OCT1* mediates the uptake and efflux of cationic metabolites in liver. Genetic variation in *OCT1* has been shown to modulate the response to metformin and other drugs [10].

Lipids: SNP rs964184 shows significant marginal epistasis for multiple lipid traits: Apolipoprotein B, cholesterol, and triglycerides with substantial marginal epistasis effects ($\frac{h^2_{gxg,t}}{h^2_{gwas,t}} = 5.14, 7.83$, and 0.59 respectively). This variant lies in the 3' UTR region of the *ZPR1* gene that encodes a zinc finger protein known to play a regulatory role in cell proliferation and signal transduction [11]. The promoter region of *ZPR1* is known to be bound by transcription factors that play a role in insulin sensitivity, cholesterol metabolism, and obesity. rs964184, as well as other variants in *ZPR1*, have been found to be associated with serum LDL-C [12], HDL-C [13], triglyceride levels and risk for coronary artery disease (CAD) [14] in diverse populations. A regulatory role for rs964184 has been suggested based on its location in a DNaseI hypersensitive region and its overlap with an enhancer that is active in tissues relevant for lipid biology [15]. Further, rs964184 has been associated with DNA methylation of a CpG site in the promoter region of the *APOA5* gene [16], potentially explaining the association between DNA methylation level at this site and triglyceride levels [17]. Integrative analyses of genotype and gene expression data have shown this SNP to play a regulatory role: cis-eQTL for *ZPR1*, a trans-eQTL for *FADS1* [18], and a trans-pQTL for *APOE* [19] (Supplementary Table 8). A previous study found evidence for genetic interaction effects on plasma lipid levels between SNPs in *ZPR1* and *APOE* [20] which is interesting given rs964184 is a trans-pQTL for *APOE* [19]. Further, mediation analyses revealed that a substantial proportion of the effect of rs964184 on HDL-C and triglycerides is mediated through its trans association with *PPM1B* and *YPEL5* [21].

6 Estimating GWAS heritability

Assuming the genotypes at the target SNP have been standardized so that $\mathbb{E}[x_{:t}] = 0$ and $Var(x_{:t}) = 1$, the variance explained by additive effects tagged by SNP t is given by:

$$\begin{aligned}\sigma^2_{gwas,t} &\equiv Var(x_{i,t}\beta_t) \\ &= \beta_t^2 Var(x_{i,t}) \\ &= \beta_t^2\end{aligned}$$

To estimate the variance explained given the GWAS estimate $\hat{\beta}_t$ of β_t (where $\hat{\beta}_t$ is the GWAS effect estimate of the standardized genotype at SNP t), we use the plug-in estimator: $\widehat{\sigma^2_{gwas,t}} = \hat{\beta}_t^2$. Using the Delta Method, we can estimate the variance of $\widehat{\sigma^2_{gwas,t}}$ as $2|\hat{\beta}_t|\widehat{SE(\hat{\beta}_t)}$. When the phenotype is standardized (as is the default assumption in our analyses), $\sigma^2_{gwas,t} = h^2_{gwas,t}$. Therefore we use these two notations interchangeably.

7 Including covariates

We can extend each of our models to include covariates as follows:

$$\boldsymbol{y} = \boldsymbol{W}\boldsymbol{\alpha} + \sum_k \boldsymbol{Z}_k\boldsymbol{\beta}_k + \boldsymbol{\epsilon} \tag{13}$$

Here $\boldsymbol{W}$ is a $N \times C$ matrix of covariates while $\boldsymbol{\alpha}$ is a vector of fixed effects of length C. Matrix $\boldsymbol{Z}_k$ can represent genotype or GxE matrices. In this setting, we need to solve the following normal equations to estimate the variance components.

$$\begin{bmatrix} \boldsymbol{T} & \boldsymbol{b} \\ \boldsymbol{b}^T & N-C \end{bmatrix} \begin{bmatrix} \sigma_1^2 \\ \vdots \\ \sigma_k^2 \\ \sigma_e^2 \end{bmatrix} = \begin{bmatrix} \boldsymbol{c} \\ \boldsymbol{y}^T\boldsymbol{V}\boldsymbol{y} \end{bmatrix} \tag{14}$$

Here $\boldsymbol{V} = \boldsymbol{I}_N - \boldsymbol{W}(\boldsymbol{W}^T\boldsymbol{W})^{-1}\boldsymbol{W}^T$ and $\boldsymbol{T}$ is a $K \times K$ matrix where $T_{k,l} = tr(\boldsymbol{K}_k\boldsymbol{V}\boldsymbol{K}_l\boldsymbol{V})$, and b is a vector of length K where $b_k = tr(\boldsymbol{V}\boldsymbol{K}_k)$, and $\boldsymbol{c}$ is a vector of length K where $c_k = \boldsymbol{y}^T\boldsymbol{V}\boldsymbol{K}_k\boldsymbol{V}\boldsymbol{y}$, $\boldsymbol{K}_k = \frac{\boldsymbol{Z}_k\boldsymbol{Z}_k^T}{M}$ where M is the number of column in Z_k. Commonly, the number of covariates C is small (tens to hundreds) so that including covariates does not significantly affect the computational cost. The cost of computing the elements of the normal equations 14 includes the cost of inverting $\boldsymbol{W}^T\boldsymbol{W}$ which is a $C \times C$ matrix and multiplying $\boldsymbol{W}$ by a real-valued vector of length N can be computed in $\mathcal{O}(C^3 + NC)$.

8 Proofs

Lemma 1.

$$(\tilde{\sigma}_g^2, \tilde{\sigma}_{gxg,t}^2, \tilde{\sigma}_e^2) = argmin_{(\sigma_g^2, \sigma_{gxg,t}^2, \sigma_e^2)} ||\boldsymbol{y}\boldsymbol{y}^T - \left(\sigma_g^2 \boldsymbol{K}_1 + \sigma_{gxg,t}^2 \boldsymbol{K}_{2,t} + \sigma_e^2 \boldsymbol{K}_3\right)||_F^2 \tag{15}$$

satisfies the following normal equations:

$$\boldsymbol{T}\boldsymbol{\sigma^2} = \boldsymbol{q} \tag{16}$$

where $\boldsymbol{K}_1 = \frac{1}{M}\boldsymbol{X}\boldsymbol{X}^T$, $\boldsymbol{K}_{2,t} = \frac{1}{M-1}\boldsymbol{E}_t\boldsymbol{E}_t^T$ *and* $\boldsymbol{K}_3 = \boldsymbol{I}_N$, $\boldsymbol{T}$ *is a* 3×3 *matrix with entries* $T_{kl} = tr(\boldsymbol{K}_k\boldsymbol{K}_l), k, l \in \{1, 2, 3\}$, $tr()$ *denotes the trace of the matrix, and* $\boldsymbol{q}$ *is a* 3*-vector with entries* $c_k = \boldsymbol{y}^T\boldsymbol{K}_k\boldsymbol{y}$.

Proof. Using the definition of the Frobenius norm of a matrix $\boldsymbol{A}$ ($||\boldsymbol{A}||_F = \sqrt{tr[\boldsymbol{A}\boldsymbol{A}^T]}$), the above equation can be re-written as:

$$(\tilde{\sigma}_g^2, \tilde{\sigma}_{gxg,t}^2, \tilde{\sigma}_e^2) = argmin_{(\sigma_g^2, \sigma_{gxg,t}^2, \sigma_e^2)} tr[(\boldsymbol{y}\boldsymbol{y}^T - \left(\sigma_g^2\boldsymbol{K}_1 + \sigma_{gxg,t}^2\boldsymbol{K}_{2,t} + \sigma_e^2\boldsymbol{K}_3\right))(\boldsymbol{y}\boldsymbol{y}^T - \left(\sigma_g^2\boldsymbol{K}_1 + \sigma_{gxg,t}^2\boldsymbol{K}_{2,t} + \sigma_e^2\boldsymbol{K}_3\right))^T]$$

Let $\boldsymbol{\theta} := [\sigma_g^2, \sigma_{gxg,t}^2, \sigma_e^2]$. Then

$$\partial f(\boldsymbol{\theta})/\partial\sigma_g^2 = 0$$
$$\Rightarrow \sigma_g^2 tr(\boldsymbol{K}_1^2) + \sigma_{gxg}^2 tr(\boldsymbol{K}_1\boldsymbol{K}_{2,t}) + \sigma_e^2 tr(\boldsymbol{K}_1\boldsymbol{K}_3) = \boldsymbol{y}^T\boldsymbol{K}_1\boldsymbol{y} \tag{17}$$

Thus, the minimizer of $f(\boldsymbol{\theta})$ must satisfy Equation 17:

$$\tilde{\sigma}_g^2 tr(\boldsymbol{K}_1^2) + \tilde{\sigma}_{gxg}^2 tr(\boldsymbol{K}_1\boldsymbol{K}_{2,t}) + \tilde{\sigma}_e^2 tr(\boldsymbol{K}_1\boldsymbol{K}_3) = \boldsymbol{y}^T\boldsymbol{K}_1\boldsymbol{y} \tag{18}$$

Similarly, we can take the partial derivative of σ_{gxg}^2 and σ_e^2 to obtain:

$$\tilde{\sigma}_g^2 tr(\boldsymbol{K}_1\boldsymbol{K}_{2,t}) + \tilde{\sigma}_{gxg}^2 tr(\boldsymbol{K}_{2,t}^2) + \tilde{\sigma}_e^2 tr(\boldsymbol{K}_{2,t}\boldsymbol{K}_3) = \boldsymbol{y}^T\boldsymbol{K}_{2,t}\boldsymbol{y} \tag{19}$$
$$\tilde{\sigma}_g^2 tr(\boldsymbol{K}_1\boldsymbol{K}_3) + \tilde{\sigma}_{gxg}^2 tr(\boldsymbol{K}_{2,t}\boldsymbol{K}_3) + \tilde{\sigma}_e^2 tr(\boldsymbol{K}_3^2) = \boldsymbol{y}^T\boldsymbol{K}_3\boldsymbol{y} \tag{20}$$

For simplicity, we can compactly rewrite the above system of equations as:

$$\boldsymbol{T}\boldsymbol{\sigma^2} = \boldsymbol{q} \tag{21}$$

where $\boldsymbol{T}$ is a 3×3 matrix with entries $T_{kl} = tr(\boldsymbol{K}_k\boldsymbol{K}_l), k, l \in \{1, 2, 3\}$, $tr()$ denotes the trace of the matrix, and $\boldsymbol{q}$ is a 3-vector with entries $c_k = \boldsymbol{y}^T\boldsymbol{K}_k\boldsymbol{y}$. □

Lemma 2. *Let* $\boldsymbol{z} \sim \mathcal{N}(\boldsymbol{0}, \boldsymbol{C})$*.* $Cov\left[\boldsymbol{z}^T\boldsymbol{A}\boldsymbol{z}, \boldsymbol{z}^T\boldsymbol{B}\boldsymbol{z}\right] = 2tr(\boldsymbol{CACB})$ *for symmetric matrices* $\boldsymbol{A}$*,* $\boldsymbol{B}$*.*

Proof.

$$\text{Cov}\left[\boldsymbol{z}^{\mathrm{T}}\boldsymbol{A}\boldsymbol{z}, \boldsymbol{z}^{\mathrm{T}}\boldsymbol{B}\boldsymbol{z}\right] = \mathbb{E}\left[\boldsymbol{z}^{\mathrm{T}}\boldsymbol{A}\boldsymbol{z}\boldsymbol{z}^{\mathrm{T}}\boldsymbol{B}\boldsymbol{z}\right] - \mathbb{E}\left[\boldsymbol{z}^{\mathrm{T}}\boldsymbol{A}\boldsymbol{z}\right]\mathbb{E}\left[\boldsymbol{z}^{\mathrm{T}}\boldsymbol{B}\boldsymbol{z}\right] \tag{22}$$

$$\begin{aligned}
\mathbb{E}\left[\boldsymbol{z}^{\mathrm{T}}\boldsymbol{A}\boldsymbol{z}\boldsymbol{z}^{\mathrm{T}}\boldsymbol{B}\boldsymbol{z}\right] &= \mathbb{E}\left[\left(\sum_{i,j} z_i A_{ij} z_j\right)\left(\sum_{k,l} z_k B_{kl} z_l\right)\right] \\
&= \mathbb{E}\left[\sum_{i,j,k,l} A_{ij}B_{kl}z_iz_jz_kz_l\right] \\
&= \sum_{i,j,k,l} A_{ij}B_{kl}\mathbb{E}\left[z_iz_jz_kz_l\right] && (23)\\
&= \sum_{i,j,k,l} A_{ij}B_{kl}[\mathbb{E}\left[z_iz_j\right]\mathbb{E}\left[z_kz_l\right] + \mathbb{E}\left[z_iz_k\right]\mathbb{E}\left[z_jz_l\right] + \mathbb{E}\left[z_iz_l\right]\mathbb{E}\left[z_kz_j\right]] && (24)\\
&= \sum_{i,j,k,l} A_{ij}B_{kl}[C_{ij}C_{kl} + C_{ik}C_{jl} + C_{il}C_{jk}] && (25)\\
&= \sum_{i,j,k,l} A_{ij}B_{kl}C_{ij}C_{kl} + \sum_{i,j,k,l} A_{ij}B_{kl}C_{ik}C_{jl} + \sum_{i,j,k,l} A_{ij}B_{kl}C_{il}C_{jk} \\
&= \sum_{i,j,k,l} A_{ij}C_{ij}B_{kl}C_{kl} + \sum_{i,j,k,l} A_{ij}C_{ik}B_{kl}C_{jl} + \sum_{i,j,k,l} A_{ij}C_{il}B_{kl}C_{jk} \\
&= \sum_{i,j} A_{ij}C_{ij}\sum_{k,l} B_{kl}C_{kl} + \sum_{i,j,k,l} A_{ij}C_{ik}B_{kl}C_{jl} + \sum_{i,j,k,l} A_{ij}C_{ik}B_{lk}C_{jl} && (26)\\
&= \left(\sum_{i,j} A_{ij}C_{ij}\right)\left(\sum_{k,l} B_{kl}C_{kl}\right) + \sum_{i,j,k,l} A_{ij}C_{ik}B_{kl}C_{jl} + \sum_{i,j,k,l} A_{ij}C_{ik}B_{kl}C_{jl} && (27)\\
&= tr(\boldsymbol{AC})tr(\boldsymbol{BC}) + 2\sum_{i,j,k,l} A_{ij}C_{ik}B_{kl}C_{jl} && (28)\\
&= tr(\boldsymbol{AC})tr(\boldsymbol{BC}) + 2\sum_{j,k}\left(\sum_i A_{ji}C_{ik}\right)\left(\sum_l B_{kl}C_{lj}\right) && (29)\\
&= tr(\boldsymbol{AC})tr(\boldsymbol{BC}) + 2\sum_{j,k}(\boldsymbol{AC})_{jk}(\boldsymbol{BC})_{kj} && (30)\\
&= tr(\boldsymbol{AC})tr(\boldsymbol{BC}) + 2tr(\boldsymbol{ACBC}) && (31)
\end{aligned}$$

Equation 23 follows by linearity of expectation while Equation 24 follows from an application of Isserlis' theorem and Equation 25 follows from the fact that $\boldsymbol{z} \sim \mathcal{N}(\boldsymbol{0}, \boldsymbol{C})$. Equation 26 follows by grouping factors in the first term and by interchanging indices l and k in the second term. Equation 27 follows from the fact that $\boldsymbol{B}$ is symmetric so that $B_{kl} = B_{lk}$. Equation 28 follows from the observation that the last two terms are identical. Equation 29 uses the fact that matrices $\boldsymbol{A}$ and $\boldsymbol{C}$ are symmetric. Equation 30 is the definition

of matrix multiplication and Equation 31 follows from the definition of the trace.

$$
\begin{aligned}
\mathbb{E}\left[\boldsymbol{z}^{\mathrm{T}}\boldsymbol{A}\boldsymbol{z}\right] &= \mathbb{E}\left[\sum_{i,j} z_i A_{ij} z_j\right] \\
&= \mathbb{E}\left[\sum_{i,j} A_{ij} z_i z_j\right] \\
&= \sum_{i,j} A_{ij}\mathbb{E}\left[z_i z_j\right] \\
&= \sum_{i,j} A_{ij} C_{ij} \\
&= tr(\boldsymbol{A}\boldsymbol{C})
\end{aligned}
\tag{32}
$$

Equation 22 follows by combining Equations 31 and 32. □

Supplementary Figures and Tables

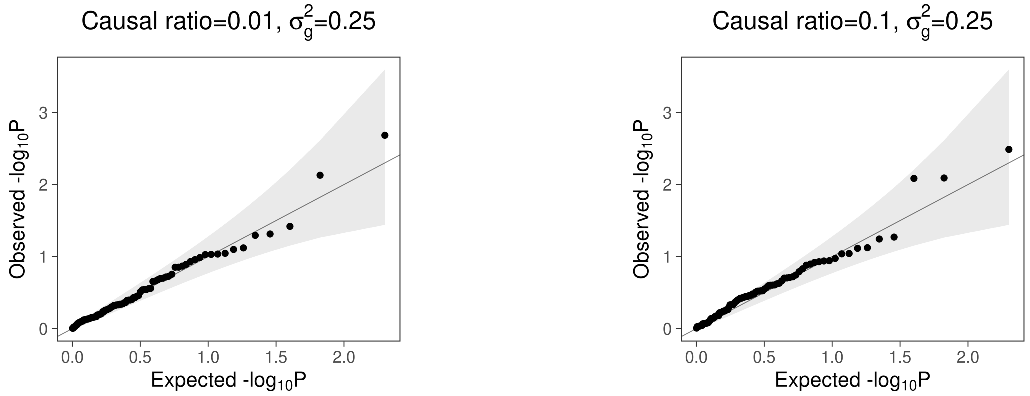

Supplementary Figure 1: **Calibration of p-values for the test of marginal epistasis under null simulations**. We simulated phenotypes with additive genetic effects but no genetic interactions. We applied FAME to 100 randomly selected SNPs from common SNPs (instead of selecting SNPs based on GWAS).

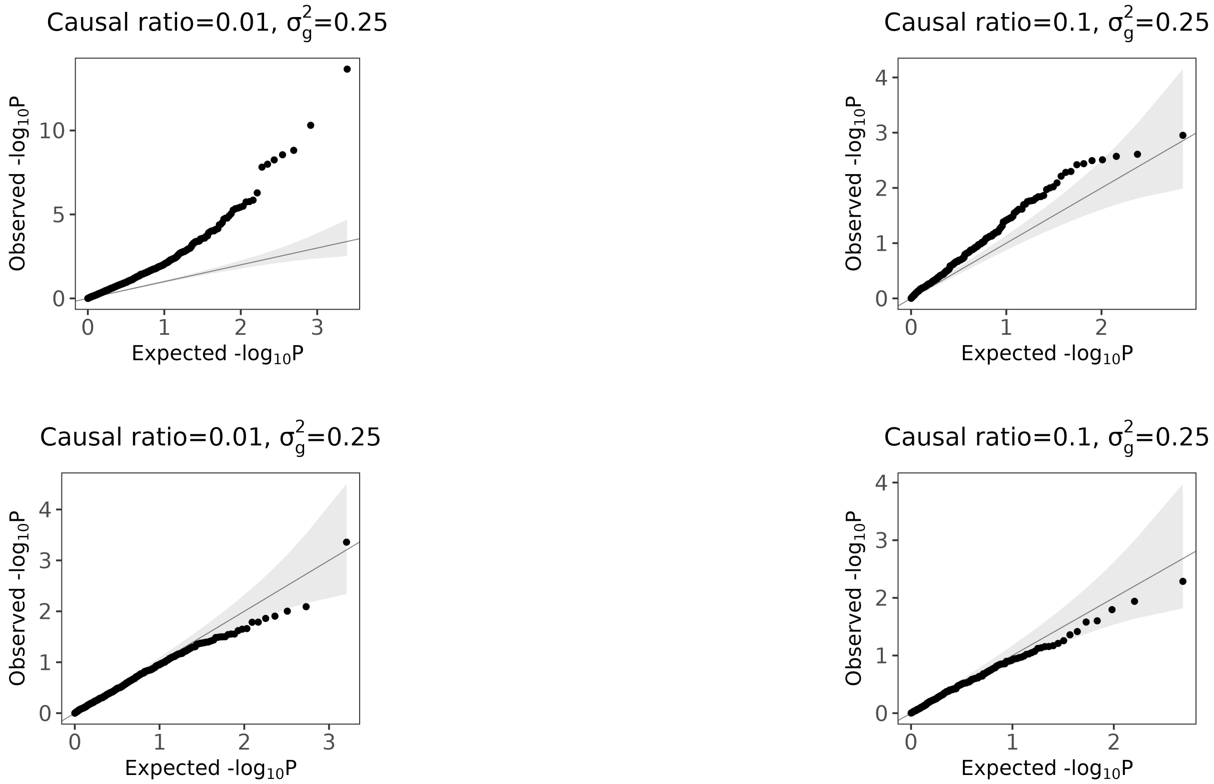

Supplementary Figure 2: **Calibration of p-values for the test of marginal epistasis based on inclusion of the LD block surrounding the target SNP**. In this experiment, we compared the results of applying FAME in two different scenarios. In scenario one, we constructed the marginal epistasis matrix by multiplying the genotypes at each target SNP with the remaining SNPs in the genome and estimated the marginal epistasis variance component and corresponding p-values (top row). In scenario two, the marginal epistasis matrix is constructed by multiplying the genotype at each target SNP with all other SNPs except those within the LD block of the target SNP, with main effects of the SNPs within the LD block regressed out. This is the standard pipeline we applied to analyze real traits (bottom row). In both experiments, the additive component (σ_g^2) for all SNPs is fit jointly with the marginal epistasis componennt $\sigma^2_{gxg,t}$. The simulated phenotype has additive but no genetic interactions. The target SNPs were chosen from the significant LD-pruned SNPs in a GWAS.

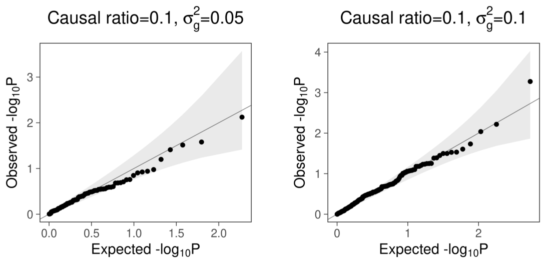

A. Polynomial covariate relationship

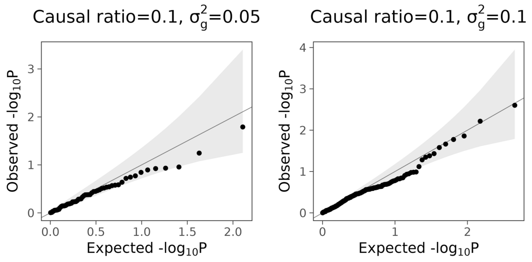

B. G-E with unobserved E

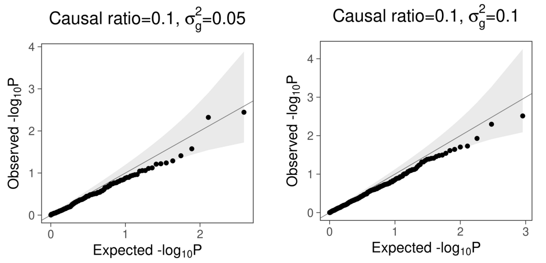

C. Heavy-tailed noise

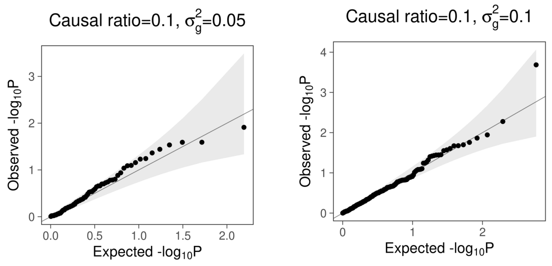

D. Heteroskedastic noise

Supplementary Figure 3: **Robustness to mis-specification**. We explored the impact of four types of model mis-specifications (Polynomial covariate relationship, G-E with unobserved E, Heavy-tailed noise, and Heteroskedastic noise) on the calibration of FAME.

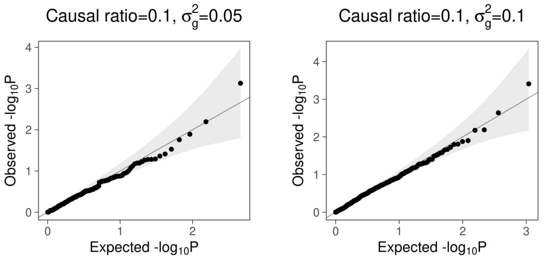

A. Large additive effects

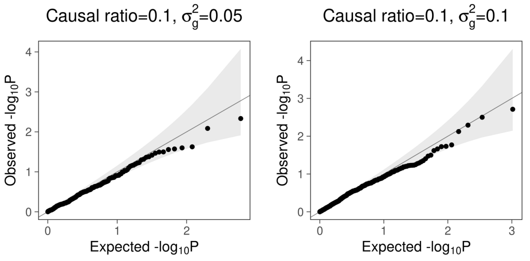

B. Imperfectly-tagged large additive effects

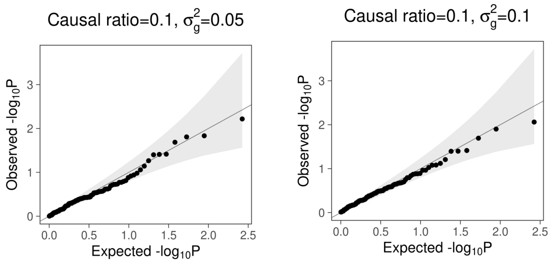

C. Imperfectly-tagged LDAK model

Supplementary Figure 4: **Robustness to mis-specification**. We explored the impact of three types of model mis-specifications (Large additive effects, Imperfectly-tagged large additive effects, and Imperfectly-tagged LDAK model) on the calibration of FAME.

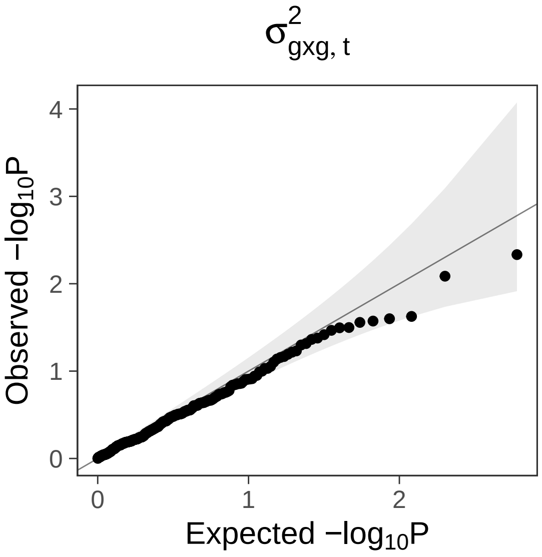

Supplementary Figure 5: **Calibration of $\hat{\sigma}^2_{gxg,t}$ under model misspecification**. We examined the calibration of p-values testing the null hypothesis that $\sigma^2_{gxg,t} = 0$ across all scenarios in which the model is misspecified and the null hypothesis of no marginal epistasis is true.

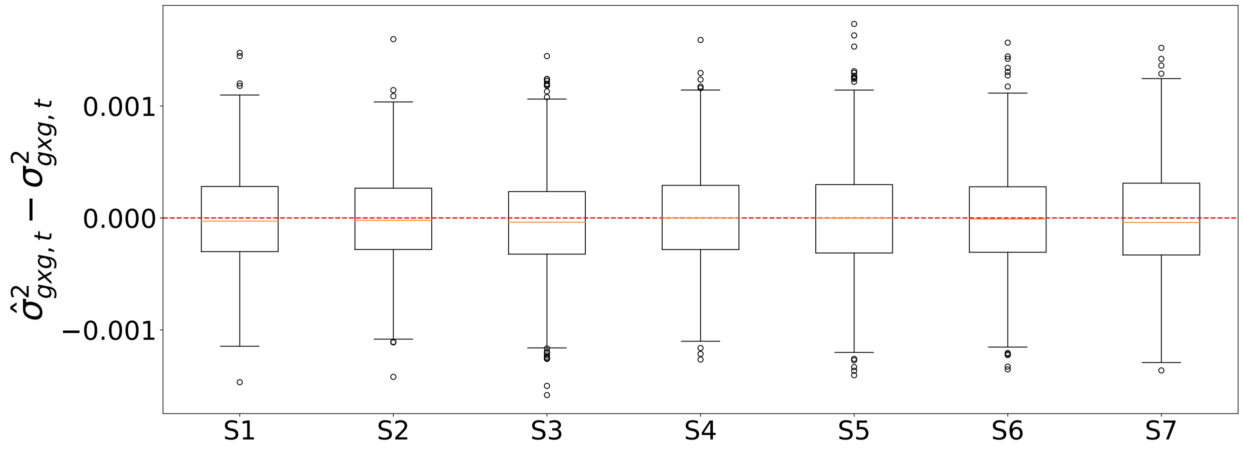

Supplementary Figure 6: **Bias under model mis-specification**. We explore the bias of FAME under the seven types of model mis-specifications. S1: Polynomial covariate relationship, S2: G-E with unobserved E, S3: Heavy-tailed noise, S4: Heteroskedastic noise, S5: Large additive effects, S6: Imperfectly-tagged large additive effects, S7: Imperfectly-tagged LDAK model. Across all the settings, FAME is relatively unbiased (magnitude of bias $< 1 \times 10^{-4}$).

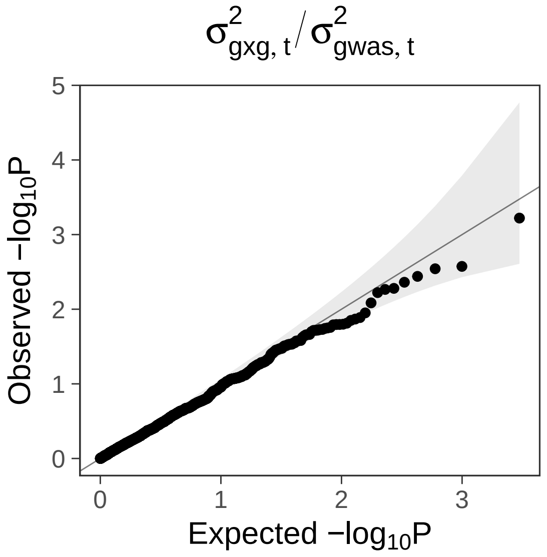

(a) Correctly specified model

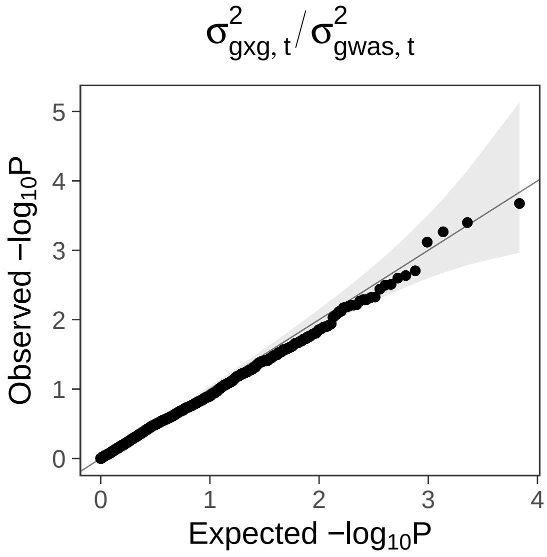

(b) Mis-specified model

Supplementary Figure 7: **Calibration of the ratio** $\frac{\hat{\sigma}^2_{gxg,t}}{\hat{\sigma}^2_{gwas,t}}$. We examined the calibration of estimates of the ratio: $\frac{\hat{\sigma}^2_{gxg,t}}{\hat{\sigma}^2_{gwas,t}}$ (a) under simulations where the model is correctly specified with causal ratio 0.1 and heritability 0.05 on *UKBB-small* data; and (b) simulations that consider model misspecifications.

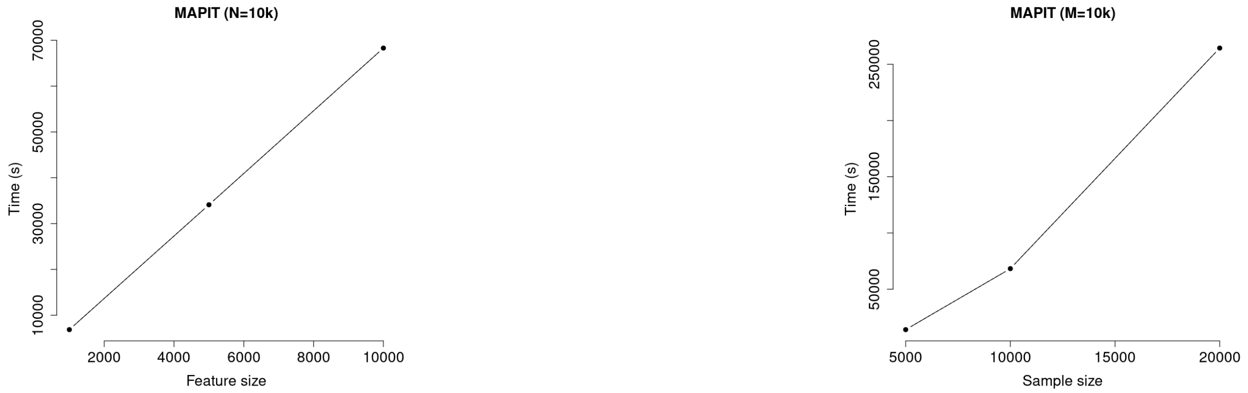

Supplementary Figure 8: **Runtime for MAPIT**. We tested MAPIT on the UKBB whole-genome array data by fixing the sample size to be 10K and varying the number of SNPs (left); and by fixing the number of SNPs to 10K and varying sample size (right).

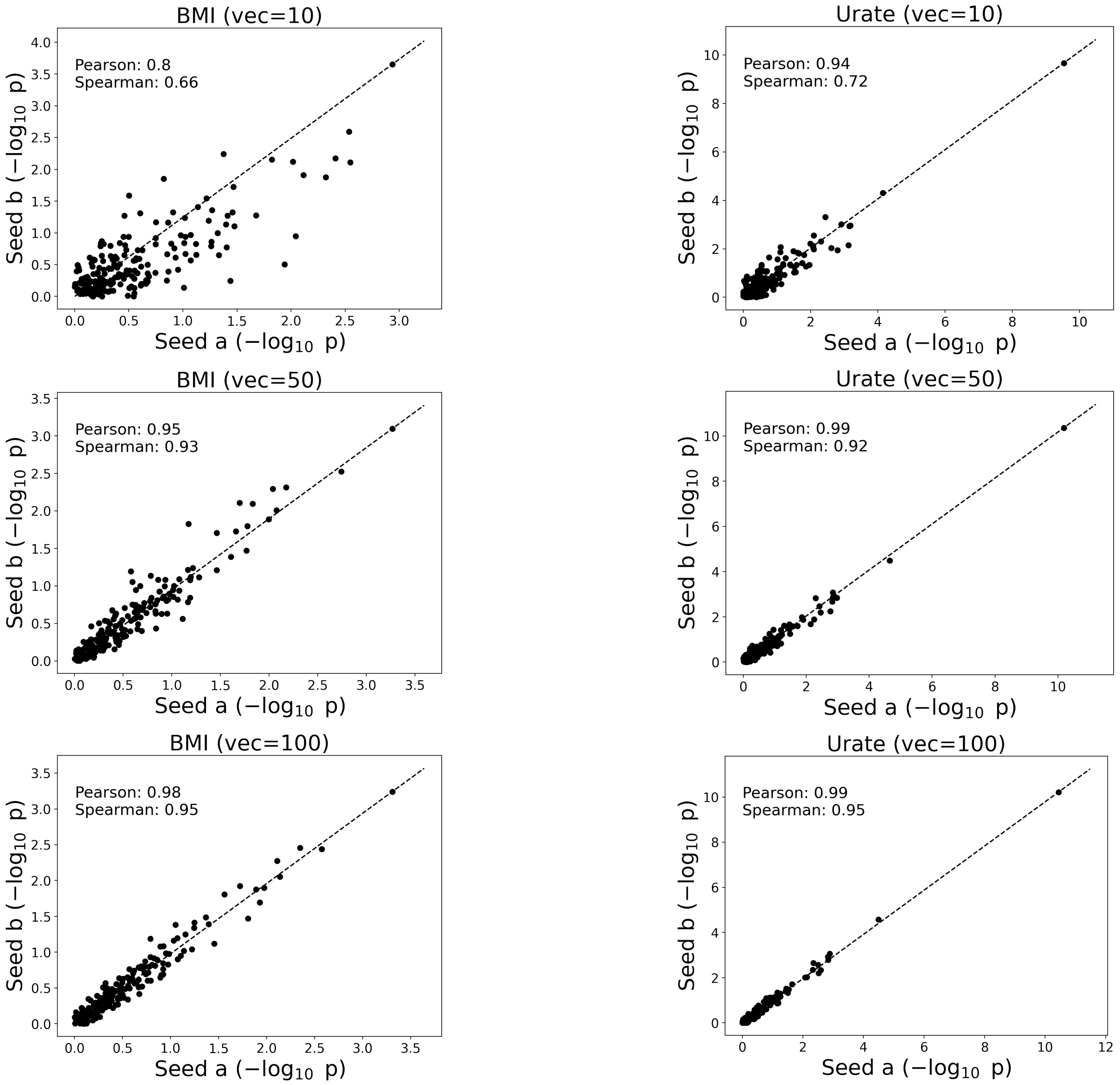

Supplementary Figure 9: **Stability of FAME p-values**. We explored the stability of the p-values estimated by FAME with varying number of random vectors. We selected two representative traits: BMI (no genome-wide significant marginal epistasis signal) and Urate (significant marginal epistasis). We chose pruned GWAS hits for each of the traits and ran FAME on these SNPs with two different seeds.

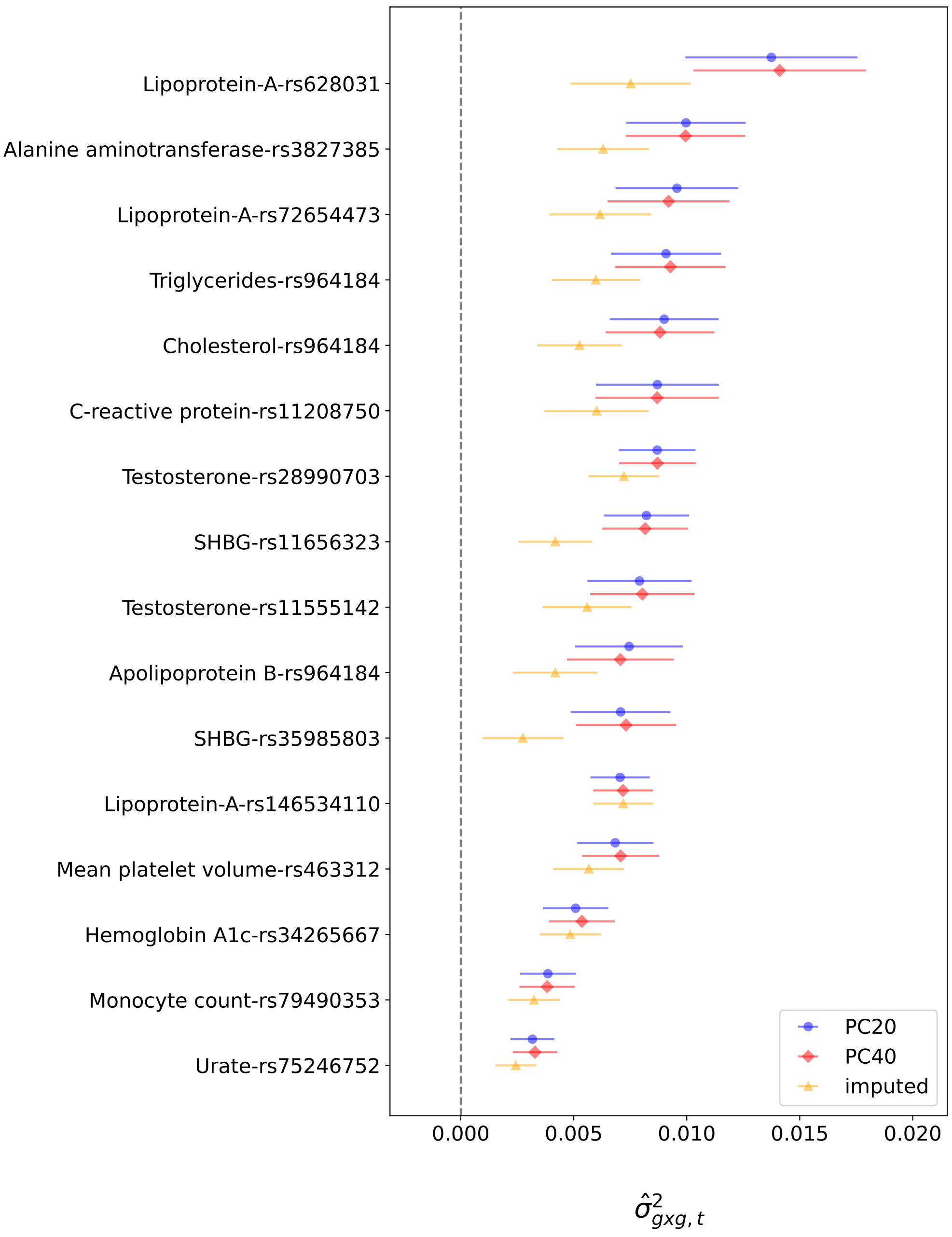

Supplementary Figure 10: **Robustness tests of marginal epistasis signals** (a) We assessed the robustness of marginal epistasis signals to population stratification. We test the trait-SNP pairs which were significant for marginal epistasis signals and repeated the test by varying the number of principal components (PC=20 vs. PC=40). (b) We assessed the robustness of marginal epistasis signals to untagged SNPs in the UKBB SNP array by testing trait-SNP pairs which were significant for marginal epistasis in the UKBB array on imputed genotypes.

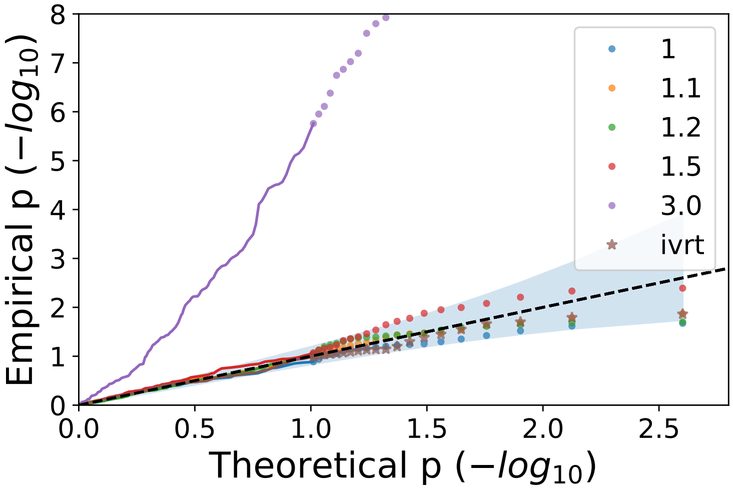

Supplementary Figure 11: **Scaling effect of simulated phenotype on marginal epistasis estimates**. We explored the impact of phenotype scale on the p-value estimated by FAME in simulations. We simulated phenotypes using genotype data from the *UKBB-small* with additive architectures (10% of the causal SNPs explaining 0.05 heritability). Next, we performed inverse rank normal transformation on the phenotype and transformed it as $sign(y) * |y|^s$, where s is a scaling factor. Finally, we randomly selected 200 GWAS hits after LD pruning as the marginal epistasis candidate and applied FAME to estimate marginal epistasis at these SNPs. The figure shows the inflation of FAME with varying scaling factors ($s = 1$ is the original unscaled phenotype). We also show the results applied to the phenotype after inverse rank normalization (IVRT); this transformation is invariant to scaling).

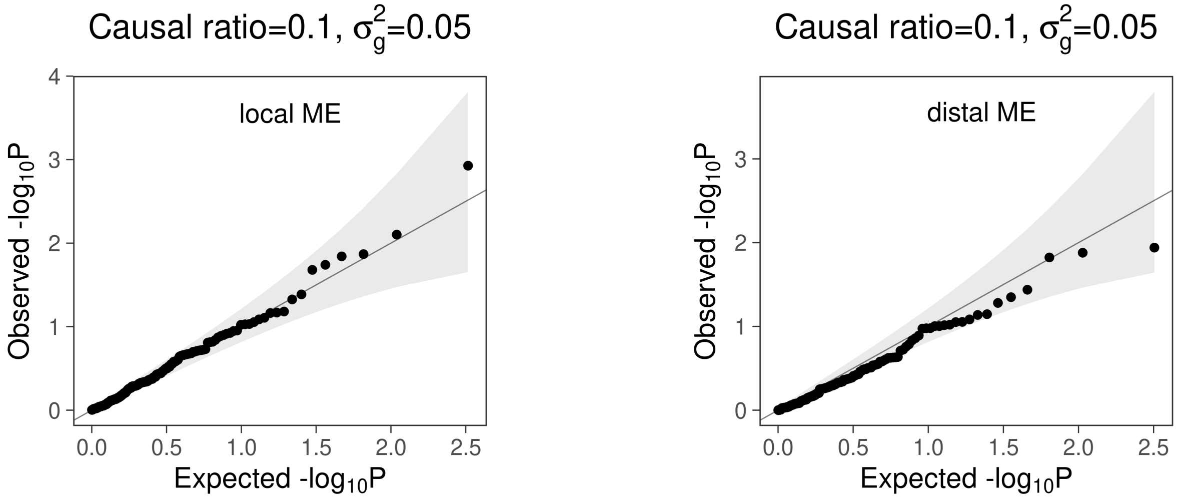

Supplementary Figure 12: **QQ-plot of tests to localize marginal epistasis in simulations**. We simulated phenotypes that consist only of linear additive effects based on *UKBB-small* (fraction of causal SNPs = 0.01 and heritability= 0.05). The target SNPs were selected to be significant SNPs obtained using a GWAS. The figure on the left shows the QQ-plot of test for marginal epistasis that lies on the same chromosome as the target SNP (with the LD block around the target SNP removed) (*local*). The figure on the right shows the QQ-plot of a test for marginal epistasis effects that lie on a different chromosome than the target SNP (*distal*). No significant marginal epistasis signals ($p < 5 \times 10^{-8}$) were found across all the settings.

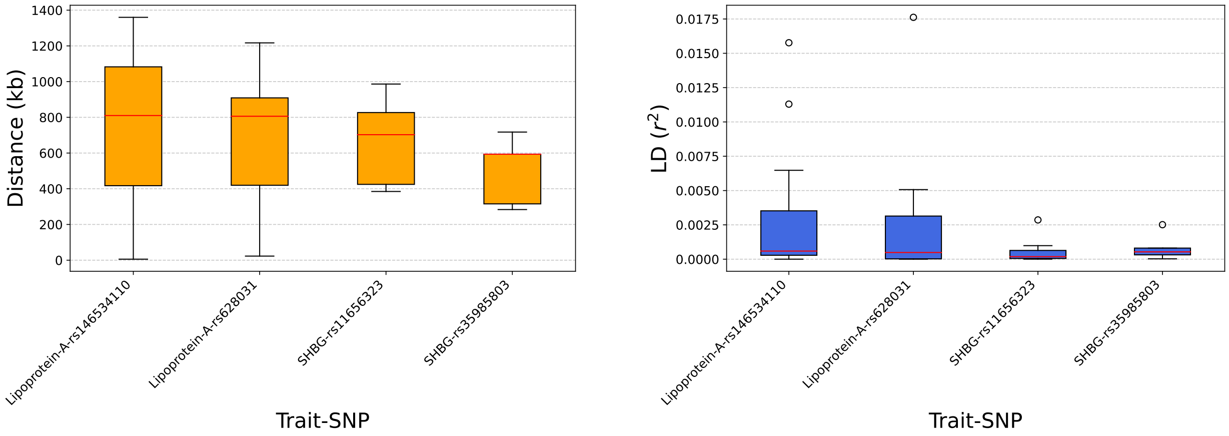

Supplementary Figure 13: **Statistics for the significant SNP pairs where the interactive SNP is located on the same chromosome as the target SNP**. We report the physical distance (left), and LD (r^2) (right) between the interactive SNPs and the target SNPs for the significant pairwise interactions across four trait-SNP pairs with significant marginal epistasis (LipoA-rs628031, LipoA-rs146534110, SHBG-rs11656323, and SHBG-rs35985803).

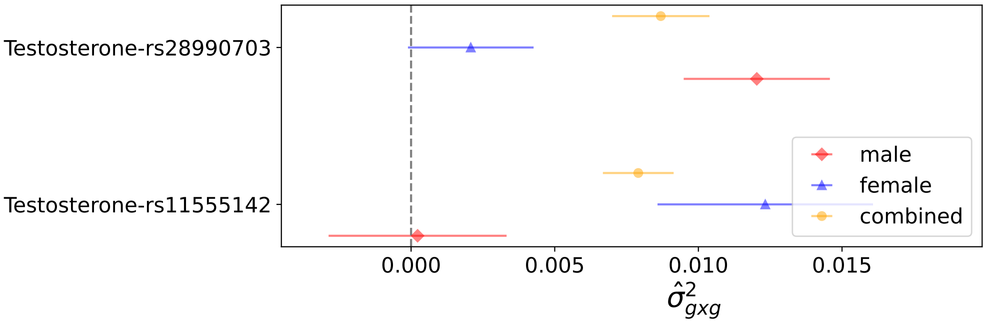

Supplementary Figure 14: **Comparison of sex-specific marginal epistasis estimates for testosterone**. We performed sex-specific marginal epistasis analysis for testosterone with 123, 339 females and 128, 287 males in the unrelated white British individuals in UKBB, and compared the corresponding marginal epistasis estimates to those from the overall (combined) analysis. We plot the point estimates and 95% CI. Both pairs demonstrate evidence for significant sex-specific difference (Welch's t-test $p < 0.05/16$).

α	0.5	0.1	0.05	0.01	0.005	λ_{gc}	CI(λ_{gc})
Polynomial covariate relationship	1.05	1.02	0.88	1.10	0.55	1.11	(0.874, 1.456)
G-E with unobserved E	0.97	0.45	0.69	0.69	0.69	0.95	(0.669, 1.287)
Heavy-tailed noise	0.93	0.71	0.68	0.62	0.93	0.82	(0.607, 1.002)
Heteroskedastic noise	0.98	1.08	1.29	0.54	0.54	0.96	(0.740, 1.232)
Large additive effects	1.02	0.89	0.77	0.77	0.77	1.03	(0.855, 1.194)
Imperfectly-tagged large additive effects	0.97	0.82	0.66	0.73	0.73	0.92	(0.766, 1.144)
Imperfectly-tagged LDAK model	1.02	0.79	0.98	0.75	0.0	1.05	(0.881, 1.439)

Supplementary Table 1: **Robustness of FAME under model misspecification**: We test the robustness of FAME under seven types of model misspecification. We report the ratio of observed to the expected type-I error rate at different p-value thresholds ($\alpha \in \{0.5, 0.1, 0.05, 0.01, 0.005\}$) and the genomic inflation factor (λ_{gc}). Across all types of model misspecification, the genomic inflation factor is close to 1 (the 95% CI of the inflation factor overlaps 1 across each of the settings). Similarly, the ratio of observed type-I error rate is close to the expected type-I error rate (none of the observed type-I error rate is significantly larger than the expectation across all thresholds and model misspecifications ($p < 0.05$)).

α	0.5	0.1	0.05	0.01	0.005	0.001	λ_{gc}	CI(λ_{gc})
Correctly specified model	0.94	0.95	0.86	0.60	0.67	0.67	0.88	(0.783, 0.984)
Mis-specified models	0.99	0.83	0.81	0.74	0.68	1.13	0.96	(0.890, 1.043)

Supplementary Table 2: **Calibration of tests of the hypothesis that $\sigma^2_{gxg} = 0$.** We test the calibration of p-values of tests of the hypothesis that $\sigma^2_{gxg} = 0$. We report the ratio of observed to the expected type-I error rate at different p-value thresholds ($\alpha \in \{0.5, 0.1, 0.05, 0.01, 0.005\}$) and the genomic inflation factor (λ_{gc}). In the first row, we reported these statistics under a correctly specified null model (causal ratio=0.1, $h^2 = 0.05$ with no marginal epistasis); in the second row, we report the combined statistics across all settings where the model is mis-specified. Across all types of model misspecification, the genomic inflation factor is close to 1 (the 95% CI of the inflation factor overlaps 1 across each of the settings) while none of the observed type-I error rates is significantly larger than the expectation across all thresholds ($p < 0.05$).

α	0.5	0.1	0.05	0.01	0.005	0.001	λ_{gc}	CI(λ_{gc})
Correctly specified model	0.94	0.95	0.86	0.60	0.67	0.67	0.88	(0.781, 0.982)
Mis-specified models	0.98	0.82	0.80	0.76	0.70	1.17	0.96	(0.884, 1.033)

Supplementary Table 3: **Calibration of tests of the hypothesis that $\frac{\sigma^2_{gxg}}{\sigma^2_{gwas}} = 0$.** We test the calibration of p-values of tests of the hypothesis that $\frac{\sigma^2_{gxg}}{\sigma^2_{gwas}} = 0$. We report the ratio of observed to the expected type-I error rate at different p-value thresholds ($\alpha \in \{0.5, 0.1, 0.05, 0.01, 0.005\}$). In the first row, we report these statistics under a correctly specified null model (causal ratio=0.1, $h^2 = 0.05$ with no marginal epistasis); in the second row, we report the combined statistics across all settinsg where the model is mis-specified. Across all types of model misspecification, the genomic inflation factor is close to 1 (the 95% CI of the inflation factor overlaps 1 across each of the settings) while none of the observed type-I error rates is significantly larger than the expectation across all thresholds ($p < 0.05$).

Trait	SNP ID	p-value
Alanine aminotransferase	rs3827385	0.30
Apolipoprotein B	rs964184	0.75
C-reactive protein	rs11208750	0.60
Cholesterol	rs964184	0.64
Hemoglobin A1c	rs34265667	0.60
Lipoprotein-A	rs628031	0.18
	rs146534110	0.59
	rs72654473	0.14
Mean platelet volume	rs463312	0.07
Monocyte count	rs79490353	0.27
SHBG	rs11656323	0.28
	rs35985803	0.36
Testosterone	rs11555142	0.63
	rs28990703	0.03
Triglycerides	rs964184	0.19
Urate	rs75246752	0.99

Supplementary Table 4: **Permutation test of marginal epistasis signals**. We permute the marginal epistasis matrix while keeping the rest of the components the same. We then run FAME on the 16 significant trait-SNP pairs reported earlier. None of the pairs are significant after permutation ($p < \frac{0.05}{16}$).

Trait	SNP ID	Sample size	$\sigma^2_{gxg,t}$ $\times 0.001$	$SE(\sigma^2_{gxg,t})$ $\times 0.001$	$p_{gxg,t}$
Alanine aminotransferase	rs3827385	70,306	12.85	3.37	1.37×10^{-4}
Monocyte count	rs79490353	70,258	3.49	1.30	7.12×10^{-3}
Mean platelet volume	rs463312	65,025	5.67	1.87	2.39×10^{-3}
Cholesterol	rs964184	56,841	11.42	3.33	6.05×10^{-4}
Triglycerides	rs964184	55,720	10.96	3.34	1.03×10^{-3}

Supplementary Table 5: **Marginal epistasis signals in the AoU dataset**. We reported the estimated marginal epistasis variance component $\sigma^2_{gxg,t}$, the corresponding standard error $SE(\sigma^2_{gxg,t})$, and the p-value significance as $p_{gxg,t}$. The sample size for each trait, excluding individuals with missing values, is reported in the Sample size field.

Supplementary Table 6: **Localizing signals of marginal epistasis**. We report point estimates of σ^2_{gxg} when the target SNP is paired with different sets of SNPs across the genome. gxg refers to the marginal epistasis estimated across the whole genome with LD block of the target SNP excluded. gxg_{local} denotes marginal epistasis effects of the target SNP paired with SNPs that lie on the same chromosome while gxg_{distal} denotes marginal epistasis effects of the target SNP paired with SNPs on different chromosomes. Entries with bold text indicates the corresponding marginal epistasis estimates were statistically significant ($p < \frac{5\times10^{-8}}{53}$)

Trait	SNP ID	gxg $\times$0.001	gxg_{local} $\times$0.001	gxg_{distal} $\times$0.001
Alanine aminotransferase	rs3827385	**9.97**	-0.22	**9.96**
Apolipoprotein B	rs964184	**7.45**	0.76	7.20
C-reactive protein	rs11208750	**8.70**	1.30	**8.53**
Cholesterol	rs964184	**9.00**	0.85	**8.71**
Hemoglobin A1c	rs34265667	**5.08**	2.75	**4.78**
Lipoprotein-A	rs628031	**13.74**	**13.47**	4.88
	rs146534110	**7.05**	**15.65**	**5.86**
	rs72654473	**9.57**	0.45	**9.62**
Mean platelet volume	rs463312	**6.83**	1.23	**6.85**
Monocyte count	rs79490353	**3.86**	1.18	3.70
SHBG	rs11656323	**8.21**	**5.16**	**7.29**
	rs35985803	**7.07**	**5.81**	5.56
Testosterone	rs11555142	**7.91**	1.36	**7.80**
	rs28990703	**8.69**	**3.18**	**8.44**
Triglycerides	rs964184	**9.08**	0.68	**9.22**
Urate	rs75246752	**3.17**	1.89	**3.24**

Trait	SNP ID	λ_{gc}	# of hits	
			Local	Distal
Alanine aminotransferase	rs3827385	1.05	0	0
Apolipoprotein B	rs964184	1.04	0	0
C-reactive protein	rs11208750	1.04	0	0
Cholesterol	rs964184	1.05	0	0
Hemoglobin A1c	rs34265667	1.08	0	0
Lipoprotein-A	rs628031	1.02	21	0
	rs146534110	1.24	23	0
	rs72654473	1.06	0	1
Mean platelet volume	rs463312	1.09	0	0
Monocyte count	rs79490353	1.11	0	0
SHBG	rs11656323	1.12	9	0
	rs35985803	1.06	5	0
Testosterone	rs11555142	1.06	0	0
	rs28990703	1.13	0	0
Triglycerides	rs964184	1.06	0	0
Urate	rs75246752	1.14	0	0

Supplementary Table 7: **Pairwise interaction analysis**. We perform a GxGWAS where we test for association of the phenotype with pairs of SNPs across the genome (where one member of the pair is the target SNP showing significant marginal epistasis for the phenotype). We report the genomic inflation factor (λ_{gc})and the number of hits (after LD pruning). The number of hits is obtained from the interacting SNP pairs that are genome-wide significant ($p < 5 \times 10^{-8}/16$). The SNPs that interact with the target marginal epistasis SNP are further pruned using the same parameters as the main analysis pipeline. Additionally, SNPs within the LD block of the target SNP and the MHC region were excluded from the final results.

SNP ID	marginal epistasis trait(s)	Overlap with external datasets
rs11208750	C-reactive protein	***cis*-eQTL** for *LEPROT, LEPR*[a]
rs628031	Lipoprotein-A	***cis*-eQTL** for *SOD2, SLC22A1, RP11-288H12.3*[a]
rs146534110	Lipoprotein-A	***cis*-pQTL** for *LPA*[b]
rs11555142	Testosterone	***cis*-eQTL** for *ZKSCAN5, CNPY4, ARPC1B, GS1-259H13.2, BUD31, PILRB, CPSF4, ZNF655, ZNF394, PTCD1*[a]
rs964184	Apolipoprotein B, Cholesterol, Triglycerides	***cis*-eQTL** for *SIDT2, TAGLN, PCSK7, ZPR1, PAFAH1B2*[a]; ***trans*-eQTL** for *HDC, ABCA1, MYLIP, GATA2, CPA3, DHCR24, SQLE, SLC45A3, FADS1, ACADVL*[a]; ***trans*-pQTL** for *LRRK2, RNF122, TPMT, GALE, ARFGAP1, METTL1, EFS, NDC80, TP53I11, EDC4, WNT5A, NAP1L2, SLC5A8, KIF3A, COPS2, FAM69C, CAMP, TIGIT, BPIFA2, IL26, CRIP1, STXBP6, PEF1, UBE2D3, EFHD1, APOE, MMP3, CYP3A4, PLA2G7, CKAP2, NXPH3, GGT2, C5orf38, NPTX2, KAZALD1, SEMA6C, PARP16, SURF1, ITM2B, DCUN1D5, KCNMB3, TMCC3, PSD2, CRYZL1, PSG5, CAMP, IFIT2, APOC3*[c]
rs28990703	Testosterone	***cis*-eQTL** for *ITFG2, RHNO1, TULP3*[a]
rs79490353	Monocyte count	***cis*-eQTL** for *FLT3*[a]
rs11656323	SHBG	***cis*-eQTL** for *ACADVL, ELP5, ASGR2, DVL2*[a]
rs35985803	SHBG	***cis*-eQTL** for *CLDN7, KCTD11, ACAP1, TNFSF12, PHF23*[a]
rs463312	Mean platelet volume	***cis*-eQTL** for *NELFCD, CTSZ, TUBB1, ZNF831*[a]
rs3827385	Alanine aminotransferase	***cis*-eQTL** for *SAMM50*[a]

Supplementary Table 8: **Overlap of significant marginal epistasis loci with previously reported eQTL and pQTL loci**.

[a]Võsa, U., Claringbould, A., Westra, HJ. et al. Large-scale cis- and trans-eQTL analyses identify thousands of genetic loci and polygenic scores that regulate blood gene expression. Nat Genet 53, 1300–1310 (2021).

[b]Yao, C., Chen, G., Song, C. et al. Genome-wide mapping of plasma protein QTLs identifies putatively causal genes and pathways for cardiovascular disease. Nat Commun 9, 3268 (2018).

[c]Ferkingstad, E., Sulem, P., Atlason, B.A. et al. Large-scale integration of the plasma proteome with genetics and disease. Nat Genet 53, 1712–1721 (2021).

SNP_1	MAF_1	SNP_2	MAF_2	$\beta_{pairwise}$	$\sigma^2_{pairwise}$	$\sigma^2_{gxg,t}$	$\sigma^2_{pairwise}/\sigma^2_{gxg,t}$
rs72613567	0.28	rs738409	0.22	-2.52×10^{-2}	0.09×10^{-3}	14.8×10^{-3}	0.0059
rs58542926	0.08	rs738409	0.22	2.91×10^{-2}	0.04×10^{-3}	14.8×10^{-3}	0.0029

Supplementary Table 9: **Summary of pairwise interaction analysis for Alanine Aminotransferase.** We report the statistics for the pairwise interaction effects on alanine aminotransferase of rs738409 and each of two SNPs, rs72613567 and rs58542926. The pairwise variance explained, $\sigma^2_{pairwise}$, is calculated as $4MAF_1(1-MAF_1)MAF_2(1-MAF_2)\beta^2_{pairwise}$, where the SNP pairs are independent (LD$\approx$ 0).

Trait	Category
BMD Heel T-score	Anthropometry
Basal metabolic rate	Anthropometry
Body mass index	Anthropometry
Height	Anthropometry
Eosinophil count	Blood biochemistry
High light scatter reticulocyte count	Blood biochemistry
IGF-1	Blood biochemistry
Lymphocyte count	Blood biochemistry
Mean corpuscular hemoglobin	Blood biochemistry
Mean platelet volume	Blood biochemistry
Mean sphered cell volume	Blood biochemistry
Monocyte count	Blood biochemistry
Platelet count	Blood biochemistry
Platelet distribution width	Blood biochemistry
RBC count	Blood biochemistry
RBC distribution width	Blood biochemistry
SHBG	Blood biochemistry
Testosterone	Blood biochemistry
White blood cell count	Blood biochemistry
Alkaline phosphatase	Bone
Calcium	Bone
Apolipoprotein A	Cardiovascular
Apolipoprotein B	Cardiovascular
C-reactive protein	Cardiovascular
Cholesterol	Cardiovascular
Diastolic blood pressure	Cardiovascular
HDL cholesterol	Cardiovascular

Trait	Category
LDL direct	Cardiovascular
Lipoprotein-A	Cardiovascular
Systolic blood pressure	Cardiovascular
Triglycerides	Cardiovascular
Glucose	Diabetes
Hemoglobin A1c	Diabetes
Corneal Hysteresis	Eye
Alanine aminotransferase	Liver
Albumin	Liver
Aspartate aminotransferase	Liver
Direct bilirubin	Liver
Total bilirubin	Liver
Creatinine	Renal
Creatinine in urine	Renal
Cystatin-C	Renal
Microalbumin in urine	Renal
Phosphate	Renal
Potassium in urine	Renal
Sodium in urine	Renal
Total protein	Renal
Urate	Renal
Urea	Renal
Age first birth	Other
FEV1-FVC ratio	Other
FVC	Other
Tanning (quantitative)	Other

Supplementary Table 10: **Traits analyzed in this study**.

Supplementary References

[1] Doug Speed et al. "Improved heritability estimation from genome-wide SNPs". In: *The American Journal of Human Genetics* 91.6 (2012), pp. 1011–1021.

[2] Alkes L Price et al. "Principal components analysis corrects for stratification in genome-wide association studies". In: *Nature genetics* 38.8 (2006), pp. 904–909.

[3] Alkes L Price et al. "New approaches to population stratification in genome-wide association studies". In: *Nature reviews genetics* 11.7 (2010), pp. 459–463.

[4] Gibran Hemani et al. "Detection and replication of epistasis influencing transcription in humans". In: *Nature* 508.7495 (2014), pp. 249–253.

[5] Frank Dudbridge and Olivia Fletcher. "Gene-environment dependence creates spurious gene-environment interaction". In: *The American Journal of Human Genetics* 95.3 (2014), pp. 301–307.

[6] Andrew R Wood et al. "Another explanation for apparent epistasis". In: *Nature* 514.7520 (2014), E3–E5.

[7] Serge Sverdlov and Elizabeth A Thompson. "The epistasis boundary: Linear vs. nonlinear genotype-phenotype relationships". In: *bioRxiv* (2018), p. 503466.

[8] Shaun Purcell et al. "PLINK: a tool set for whole-genome association and population-based linkage analyses". In: *The American journal of human genetics* 81.3 (2007), pp. 559–575.

[9] Eric Boerwinkle et al. "Apolipoprotein (a) gene accounts for greater than 90% of the variation in plasma lipoprotein (a) concentrations." In: *The Journal of clinical investigation* 90.1 (1992), pp. 52–60.

[10] Yan Shu et al. "Effect of genetic variation in the organic cation transporter 1 (OCT1) on metformin action". In: *The Journal of clinical investigation* 117.5 (2007), pp. 1422–1431.

[11] Zoya Galcheva-Gargova et al. "Binding of zinc finger protein ZPR1 to the epidermal growth factor receptor". In: *Science* 272.5269 (1996), pp. 1797–1802.

[12] Tanya M Teslovich et al. "Biological, clinical and population relevance of 95 loci for blood lipids". In: *Nature* 466.7307 (2010), pp. 707–713.

[13] Sekar Kathiresan et al. "Common variants at 30 loci contribute to polygenic dyslipidemia". In: *Nature genetics* 41.1 (2009), pp. 56–65.

[14] Heribert Schunkert et al. "Large-scale association analysis identifies 13 new susceptibility loci for coronary artery disease". In: *Nature genetics* 43.4 (2011), pp. 333–338.

[15] Esteban J Parra et al. "Admixture mapping in two Mexican samples identifies significant associations of locus ancestry with triglyceride levels in the BUD13/ZNF259/APOA5 region and fine mapping points to rs964184 as the main driver of the association signal". In: *PLoS One* 12.2 (2017), e0172880.

[16] Erika L Moen et al. "Genome-wide variation of cytosine modifications between European and African populations and the implications for complex traits". In: *Genetics* 194.4 (2013), pp. 987–996.

[17] L Pfeiffer et al. "DNA methylation of lipid-related genes affects blood lipid levels". In: *Circ Cardiovasc Genet* 8.2 (2015), pp. 334–42.

[18] Chen Yao et al. "Genome-wide mapping of plasma protein QTLs identifies putatively causal genes and pathways for cardiovascular disease". In: *Nature communications* 9.1 (2018), p. 3268.

[19] Egil Ferkingstad et al. “Large-scale integration of the plasma proteome with genetics and disease”. In: *Nature genetics* 53.12 (2021), pp. 1712–1721.

[20] Magdalena Zimoń et al. “Pairwise effects between lipid GWAS genes modulate lipid plasma levels and cellular uptake”. In: *Nature communications* 12.1 (2021), p. 6411.

[21] Chen Yao et al. “Integromic analysis of genetic variation and gene expression identifies networks for cardiovascular disease phenotypes”. In: *Circulation* 131.6 (2015), pp. 536–549.
