# Supplementary material for: A biobank-scale test of marginal epistasis reveals genome-wide signals of polygenic interaction effects
Source: Nat Genet. 2025 Dec 9;57(12):3175–84. doi: 10.1038/s41588-025-02411-y (PMC12695669; doi:10.1038/s41588-025-02411-y)
Supplement: Supplementary file 2 — Reporting Summary [file 41588_2025_2411_MOESM2_ESM.pdf]

nature portfolio

Corresponding author(s): Sriram Sankararaman

Last updated by author(s): Aug 11, 2025

Reporting Summary

Nature Portfolio wishes to improve the reproducibility of the work that we publish. This form provides structure for consistency and transparency in reporting. For further information on Nature Portfolio policies, see our Editorial Policies and the Editorial Policy Checklist.

Statistics

For all statistical analyses, confirm that the following items are present in the figure legend, table legend, main text, or Methods section.

n/a	Confirmed	
☐	☒	The exact sample size (n) for each experimental group/condition, given as a discrete number and unit of measurement
☒	☐	A statement on whether measurements were taken from distinct samples or whether the same sample was measured repeatedly
☐	☒	The statistical test(s) used AND whether they are one- or two-sided<br>*Only common tests should be described solely by name; describe more complex techniques in the Methods section.*
☐	☒	A description of all covariates tested
☐	☒	A description of any assumptions or corrections, such as tests of normality and adjustment for multiple comparisons
☐	☒	A full description of the statistical parameters including central tendency (e.g. means) or other basic estimates (e.g. regression coefficient) AND variation (e.g. standard deviation) or associated estimates of uncertainty (e.g. confidence intervals)
☐	☒	For null hypothesis testing, the test statistic (e.g. F, t, r) with confidence intervals, effect sizes, degrees of freedom and P value noted<br>*Give P values as exact values whenever suitable.*
☒	☐	For Bayesian analysis, information on the choice of priors and Markov chain Monte Carlo settings
☐	☒	For hierarchical and complex designs, identification of the appropriate level for tests and full reporting of outcomes
☐	☒	Estimates of effect sizes (e.g. Cohen's d, Pearson's r), indicating how they were calculated

Our web collection on statistics for biologists contains articles on many of the points above.

Software and code

Policy information about availability of computer code

Data collection	No new data was collected for this study.
Data analysis	The code for marginal epistasis testing and estimation (FAME) is freely available at https://github.com/sriramlab/FAME . The simulator used to benchmark FAME is freely available at https://github.com/sriramlab/Simulator. MAPIT software can be found at https://github.com/lorinanthony/MAPIT.

For manuscripts utilizing custom algorithms or software that are central to the research but not yet described in published literature, software must be made available to editors and reviewers. We strongly encourage code deposition in a community repository (e.g. GitHub). See the Nature Portfolio guidelines for submitting code & software for further information.

Data

Policy information about availability of data

All manuscripts must include a data availability statement. This statement should provide the following information, where applicable:

- Accession codes, unique identifiers, or web links for publicly available datasets
- A description of any restrictions on data availability
- For clinical datasets or third party data, please ensure that the statement adheres to our policy

The UK Biobank dataset used in this study is not publicly available but can be obtained by application (https://www.ukbiobank.ac.uk/). The UK Biobank data was accessed under application number 33127. The All of Us dataset used in this study can be accessed via the public Data Browser upon approval (https://

databrowser.researchallofus.org/). The workspace namespace for All of Us for this project is aou-rw-a5bbcc47. The eQTL data examined are available at https://www.nature.com/articles/s41588-021-00913-z. The pQTL data are available at https://www.nature.com/articles/s41586-023-06812-z and https://www.nature.com/articles/s41588-021-00978-w.

Research involving human participants, their data, or biological material

Policy information about studies with human participants or human data. See also policy information about sex, gender (identity/presentation), and sexual orientation and race, ethnicity and racism.

Reporting on sex and gender	We analyzed unrelated white British individuals from the UK Biobank data. We jointly analyzed males and females while regressing out sex as a covariate in our analyses. The majority of our findings are relevant to the general population in the UK Biobank and are not gender/sex specific. We performed sex-specific analysis of marginal espistasis effects underlying serum testosterone.
Reporting on race, ethnicity, or other socially relevant groupings	Our analyses focus on white British individuals as defined in the UK Biobank.
Population characteristics	The UK Biobank recruited individuals from the UK aged between 49 and 60. Our analysis focused on the subset of unrelated white British individuals in the UK Biobank. All of Us recruited individuals 18 years of age or older from a network of recruitment sites across the USA.
Recruitment	No new data was collected for this study.
Ethics oversight	Ethics committee/IRB of UKBB gave ethical approval for collection of UKBB data (https://www.ukbiobank. ac.uk/learn-more-about-uk-biobank/about-us/ethics). Approval to use UKBB individual level in this work was obtained under application 33127 at http://www.ukbiobank.ac.uk. Ethics committee/IRB of AoU gave ethical approval for collection of AoU data (https://allofus.nih.gov/about/who-we-are/ institutional-review-board-irb-of-all-of-us-research-program). Approval to use AoU controlled tier data in this work was obtained through application at https://www.researchallofus.org.

Note that full information on the approval of the study protocol must also be provided in the manuscript.

Field-specific reporting

Please select the one below that is the best fit for your research. If you are not sure, read the appropriate sections before making your selection.

☒ Life sciences ☐ Behavioural & social sciences ☐ Ecological, evolutionary & environmental sciences

For a reference copy of the document with all sections, see nature.com/documents/nr-reporting-summary-flat.pdf

Life sciences study design

All studies must disclose on these points even when the disclosure is negative.

Sample size	For analysis of real traits, we restricted our analysis to SNPs that were present in the UK Biobank Axiom array used to genotype the UK Biobank. SNPs with greater than 1% missingness and minor allele frequency smaller than 1% were removed. Moreover, SNPs that fail the Hardy-Weinberg test at significance threshold 10–7 were removed. We restricted our study to self-reported British white ancestry individuals which are > 3rd degree relatives that is defined as pairs of individuals with kinship coefficient < 1/2(9/2) [5]. Furthermore, we removed individuals who are outliers for genotype heterozygosity and/or missingness and excluded SNPs that fall within the MHC region. Finally, we obtained a set of N = 291,273 individuals and M = 454,207 SNPs for real data analyses. We used this dataset in our analyses unless specified otherwise.
Data exclusions	We excluded individuals in the UK Biobank who were not classified as White British and removed closely related individuals (closer than third-degree relatives). These exclusions were made to reduce potential confounding due to population stratification and other unobserved factors correlated with population structure.
Replication	We performed an internal replication within UK Biobank and also an external replication of the UK Biobank results in the All of Us (AoU) where we analyzed traits that had a N >50K individuals of European ancestry in the AoU data.
Randomization	Randomization was not applicable, as this study analyzed existing observational data.
Blinding	Blinding was not applicable, as this study analyzed existing observational data and did not generate new experimental results.

Reporting for specific materials, systems and methods

We require information from authors about some types of materials, experimental systems and methods used in many studies. Here, indicate whether each material, system or method listed is relevant to your study. If you are not sure if a list item applies to your research, read the appropriate section before selecting a response.

Materials & experimental systems

n/a	Involved in the study
☒	☐ Antibodies
☒	☐ Eukaryotic cell lines
☒	☐ Palaeontology and archaeology
☒	☐ Animals and other organisms
☒	☐ Clinical data
☒	☐ Dual use research of concern
☒	☐ Plants

Methods

n/a	Involved in the study
☒	☐ ChIP-seq
☒	☐ Flow cytometry
☒	☐ MRI-based neuroimaging

Plants

Seed stocks	*Report on the source of all seed stocks or other plant material used. If applicable, state the seed stock centre and catalogue number. If plant specimens were collected from the field, describe the collection location, date and sampling procedures.*
Novel plant genotypes	*Describe the methods by which all novel plant genotypes were produced. This includes those generated by transgenic approaches, gene editing, chemical/radiation-based mutagenesis and hybridization. For transgenic lines, describe the transformation method, the number of independent lines analyzed and the generation upon which experiments were performed. For gene-edited lines, describe the editor used, the endogenous sequence targeted for editing, the targeting guide RNA sequence (if applicable) and how the editor was applied.*
Authentication	*Describe any authentication procedures for each seed stock used or novel genotype generated. Describe any experiments used to assess the effect of a mutation and, where applicable, how potential secondary effects (e.g. second site T-DNA insertions, mosiacism, off-target gene editing) were examined.*
